# Whole transcriptome analysis of bovine mammary progenitor cells by P-Cadherin enrichment as a marker in the mammary cell hierarchy

**DOI:** 10.1038/s41598-020-71179-4

**Published:** 2020-08-25

**Authors:** E. Martignani, U. Ala, P. A. Sheehy, P. C. Thomson, M. Baratta

**Affiliations:** 1 Department of Veterinary Science, University of Turin, Via Largo Braccini 2, 10095 Grugliasco, TO Italy; 2 Sydney School of Veterinary Science, The University of Sydney, 425 Werombi Road, Camden, NSW 2570 Australia; 3 School of Life and Environmental Sciences, The University of Sydney, 425 Werombi Road, Camden, NSW 2570 Australia

**Keywords:** Developmental biology, Stem cells, Zoology

## Abstract

Adult bovine mammary stem cells possess the ability to regenerate in vivo clonal outgrowths that mimic functional alveoli. Commonly available techniques that involve immunophenotype-based cell sorting yield cell fractions that are moderately enriched, far from being highly purified. Primary bovine mammary epithelial cells segregated in four different populations according to the expression of P-Cadherin and CD49f. Sorted cells from each fraction were tested for the presence of lineage-restricted progenitors and stem cells. Only cells from the CD49f^high^/P-Cadherin^neg^ subpopulation were able to give rise to both luminal- and myoepithelial-restricted colonies in vitro and generate organized outgrowths in vivo, which are hallmarks of stem cell activity. After whole transcriptome analysis, we found gene clusters to be differentially enriched that relate to cell-to-cell communication, metabolic processes, proliferation, migration and morphogenesis. When we analyzed only the genes that were differentially expressed in the stem cell enriched fraction, clusters of downregulated genes were related to proliferation, while among the upregulated expression, cluster of genes related to cell adhesion, migration and cytoskeleton organization were observed. Our results show that P-Cadherin separates mammary subpopulations differentially in progenitor cells or mammary stem cells. Further we provide a comprehensive observation of the gene expression differences among these cell populations which reinforces the assumption that bovine mammary stem cells are typically quiescent.

## Introduction

It has been well established that the cyclical development of the mammary gland in mammals represents a valuable model of the processes underlying cell differentiation for the dynamic remodeling of the organ and of its function, in this case, the production and secretion of milk. This interest was further strengthened when in 2006 two studies showed the presence of a single pluripotent cell possessed the ability to completely regenerate the functional unit of the gland, the mammary alveolus^1,2^. Further, a single cell clone has been reported to be able to differentiate in a bilayered system organized into alveolar structures connected by a common ductal system. The two cell layers are separate but contiguous throughout and consist of an inner layer of cytokeratin CK18^+^ luminal cells and an outer layer of cytokeratin CK14^+^ myoepithelial cells^2^. In subsequent years, further studies were carried out on the characterization of this niche of mammary pluripotent stem cells in the human or in laboratory animals and on the maintenance and activation processes of adult stem cells both in physiological and pathological contexts^3–6^. The purification and isolation of these cells in other species is made more difficult due to the lack of species-specific cellular information and the availability of specific antibodies for the individual species although cell isolation and characterization has been reported in the bovine, caprine, canine and feline species ^7–10^. Interestingly, among mammalians species, with respect to the development and interaction of the alveolar functional unit and the extracellular matrix of the mammary gland, the morphological development of the mammary gland in humans and ruminants is similar^11^, revealing the potential of ruminant species to serve as useful models for a better understanding of human breast development and cancer.

In the phenotypic characterization of this small stem cell population, some surface markers appear to be established and in part common among species, such as alpha-6 integrin (CD49f) and CD24^12–15^ as well as intracellular enzymes which have been evaluated for some species (i.e. absence of ALDH1 activity^16^. Furthermore, it is well accepted that this stem cell niche is localized in close association with the basal layer of the alveolus^17^.

P-Cadherin is a calcium-dependent cell–cell adhesion glycoprotein with a homeostatic function in several tissues and its name is derived from the site where this adhesion molecule was firstly characterized, the placenta^18^. It exerts a crucial role in the conservation of the structural integrity of epithelial tissues and regulates processes involved in embryonic development and maintenance of adult tissue architecture, important for cell differentiation, cell shape, cell polarity, growth and migration^19–21^.

P-Cadherin is of interest in that it plays a role both during embryonic development and during the processes of differentiation of adult tissue, maintaining its architecture through cell differentiation, polarity organization, the induction of migration and proliferation^22^.

Despite being expressed in human fetal structure^23,24^, P-Cadherin is present in several adult tissues, usually co-expressed with E-Cadherin, such as the basal layer of the epidermis, the mammary gland and the prostate, as well as the mesothelium, the ovary, the cervix, the hair follicle, and the corneal endothelium^25^.

It has been reported that P-Cadherin may be involved in the biology of mammary stem cells as its expression was observed in a cellular subpopulation with a phenotype similar to the basal epithelial cells and which co-expressed with SLUG, a transcription factor that regulates cadherin genes and the genes involved in mammary epithelial cell lineages^26^, but within the basal or myoepithelial layer where the niche of multipotent cells are believed to reside^27–29^. Moreover during the evolution of the gland, P-Cadherin is found in stem cells that give rise to the myoepithelial cell layer, termed cap cells^30,31^. In contrast, in the mature lactating gland, P-Cadherin is expressed in human alveolar cells, the units responsible for milk protein production^32^.

To date, however, there is no functional evidence of the role of P-Cadherin for the generation of a mammary alveolus and normal function. While it is interesting to confirm the possible identification of a new marker linked to mammary stem cell populations, in particular for a species where few molecular markers are available, it is necessary to examine the cellular processes that occur in this cell niche in the presence of P-Cadherin.

The aim of this research is to demonstrate the utility of P-Cadherin in the characterization of the cellular phenotype of adult stem cells in the mammary gland and to report which genes are activated in this role during the process of mammary differentiation through transcriptome analysis.

## Results

### Frequencies of the mammary epithelial subpopulations

**Figure 1:**
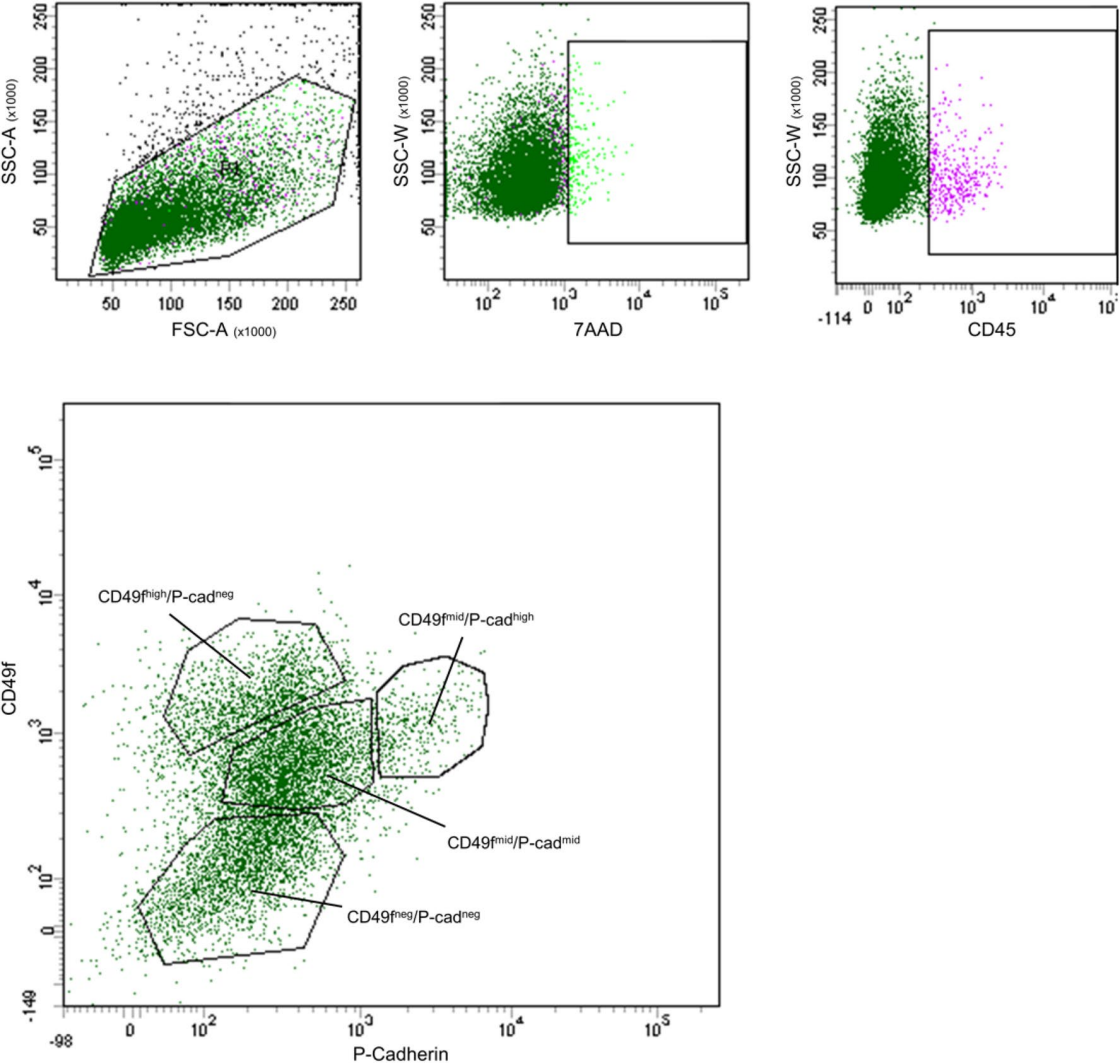
Sorting strategy for separating mammary subpopulations. The figure shows representative scatterplots of a sample of bovine mammary epithelial cells and the general sorting strategy adopted. A broad gate was set for the physical parameters, then events that were positive for 7-AAD (dead cells) and CD45 + (leucocytes) were excluded from the population of interest. Cells were then separated according to the expression levels of CD49f and P-Cadherin.

The expression of CD49f. and P-Cadherin were analyzed by flow cytometry in mammary epithelial cells recovered from eight Piedmontese heifers. After exclusion of dead cells, four subpopulations were identified according to the expression levels of both markers (Fig. 1). A double negative population (CD49f^neg^/P-Cadherin^neg^), a highly CD49f positive/P-Cadherin negative population (CD49f^high^/P-Cadherin^neg^), a medium CD49f/medium P-Cadherin positive population (CD49f^mid^/P-Cadherin^mid^) and a medium CD49f/highly P-Cadherin positive population (CD49f^mid^/P-Cadherin^high^) were identified in all samples analyzed. The distribution of these populations shows variability among samples: CD49f^neg^/P-Cadherin^neg^ ranged from 5 to 30.7%, CD49f^high^/P-Cadherin^neg^ ranged from 10.3 to 52.6%, CD49f^mid^/P-Cadherin^mid^ ranged from 8.6 to 40.6% and CD49f^mid^/P-Cadherin^high^ ranged from 3.9 to 40%.

The expression of CD45 was also evaluated and CD45^+^ cells accounted for less than 5% of the starting population in all analyzed samples.

### Distribution of mammary progenitor cells among sorted fractions

As we have previously shown, in adult bovine mammary tissue, both stem cells and committed unipotent progenitor cells can be observed^38^. A clonogenic assay such as the CFC allows for the detection of highly proliferating cells that are committed to either a myoepithelial or a luminal phenotype. We carried out the CFC assay with the four sorted fractions in order to investigate whether the progenitors that were present in the mammary tissue were associated with one or more of the phenotypes that we identified and therefore in which of the subpopulations they segregated.

**Figure 2:**
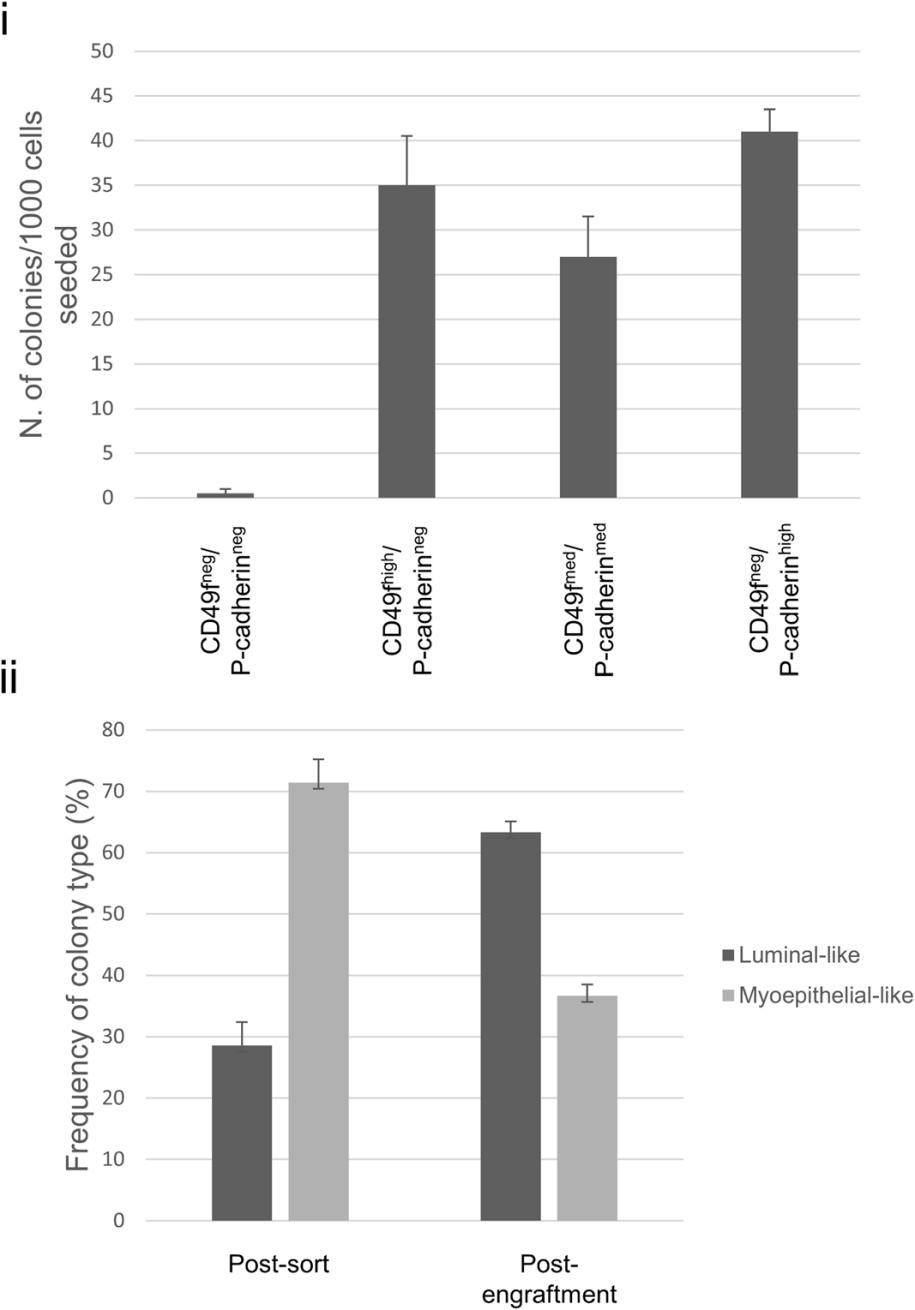
Frequency distribution of mammary progenitors among sorted fractions. (**i**) frequency of mammary progenitors in the four isolated cell fraction is expressed as number of colonies per 1,000 cells seeded (*n* = 3) ± s.e. (**ii**) relative frequency of luminal and myoepithelial colonies within the CD49f^high^/P-Cadherin^neg^ subpopulation when cells were seeded immediately after sorting and after cells of the same fraction were engrafted in NOD/SCID mice.

In all the dishes where cells from the CD49f^neg^/P-Cadherin^neg^ fractions were seeded, the overall frequency of colonies was lower than 0.05%, while within cell populations of the other fractions were seeded, the colony frequency ranged from 2.7 to 4.1% (Fig. 2i). Interestingly, when the colonies were scored according to their morphology as previously described [both in the CD49f^mid^/P-Cadherin^mid^ and in the CD49f^mid^/P-Cadherin^high^ fractions], no myoepithelial-like colonies could be observed and only luminal-like colonies were apparent. In the CD49f^igh^/P-Cadherin^neg^ fraction however, myoepithelial-like colonies could be observed and their relative frequency was 71.4 ± 3.8% (number of myoepithelial colonies / total number colonies × 100 ± s.e.) (Fig. 2ii).

### Mammary epithelial stem cells segregate in the CD49f^high^*/P-Cadherin*^neg^ subpopulation

While the CFC assay is efficient in detecting highly proliferating cells of different lineages (i.e. committed progenitors), it is not suitable to detect cells that retain stem-like properties. Therefore cells from different subpopulations were xenografted into immunocompromised mice as an in vivo model that has been previously described^3,33^. In a xenotrasplantation model, only mammary stem cells are able to engraft and regenerate outgrowths that mirror ducts and acini found in the bovine mammary tissue. In previous experiments, it was established that 7.5 × 10^5^ cells were an optimal number to obtain a sufficient number of outgrowths to enable analysis. Therefore for each sorted subpopulation, the number of cells that were engrafted was calculated as equal to the frequency of the subpopulation measured when sorting, applied to a 7.5 × 10^5^ total cell number.

Three bovine mammary samples were selected and for each subpopulation, multiple gels were prepared. Two to three gels were engrafted into each kidney for a total of five to six gels per bovine sample/experiment. When recovered, two to three gels per subpopulation were dissociated and seeded in CFC assays to assess the progenitor cell content of the xenografts, while the remaining gels were paraffin-embedded and sectioned for immunohistological analysis.

**Figure 3:**
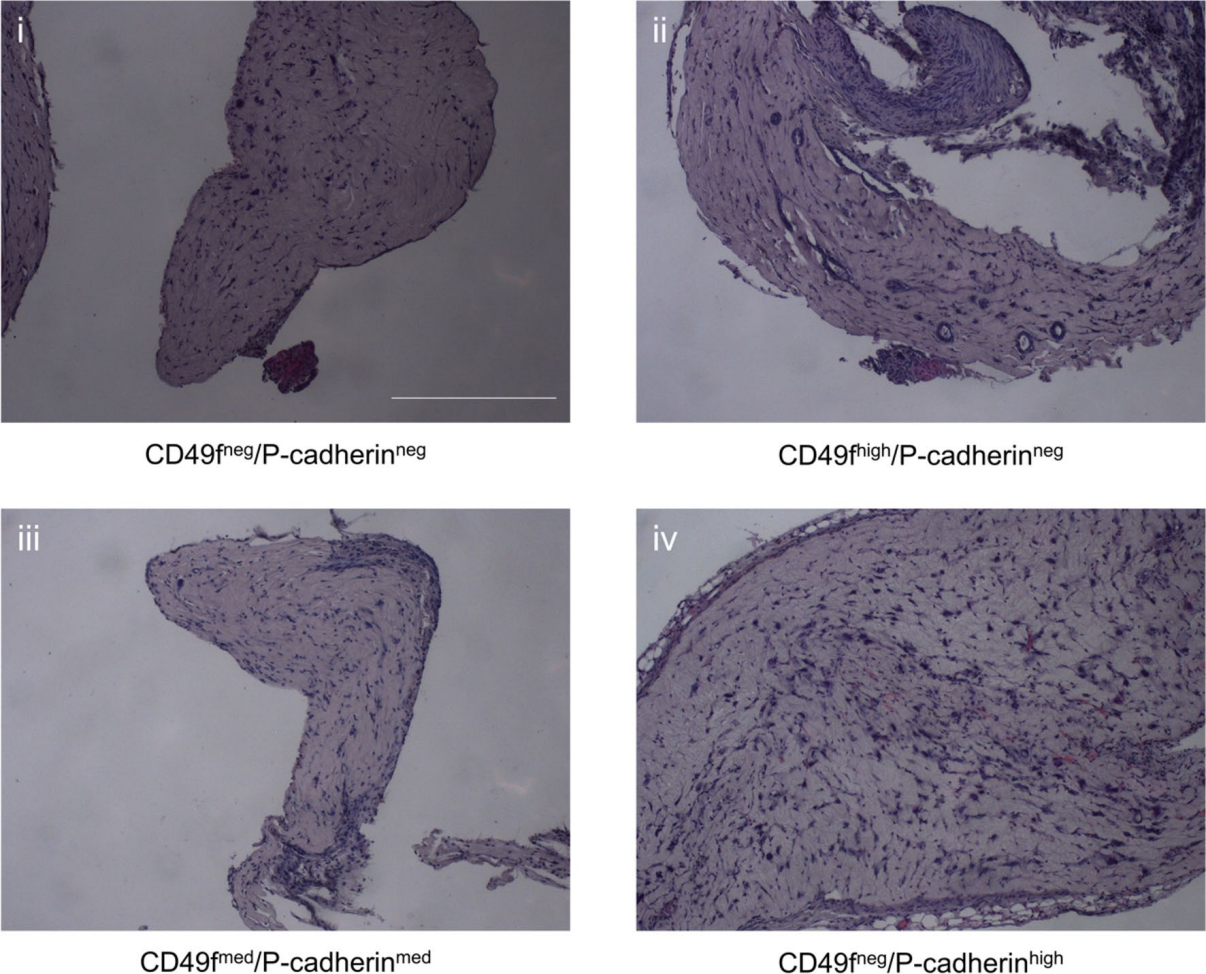
Histological sections of xenografted gels. Representative images of xenografted gels from each of the mammary epithelial cell subpopulations after 4 weeks of in vivo engraftment. Hematoxylin staining, scale bar is 250 µm.

**Figure 4:**
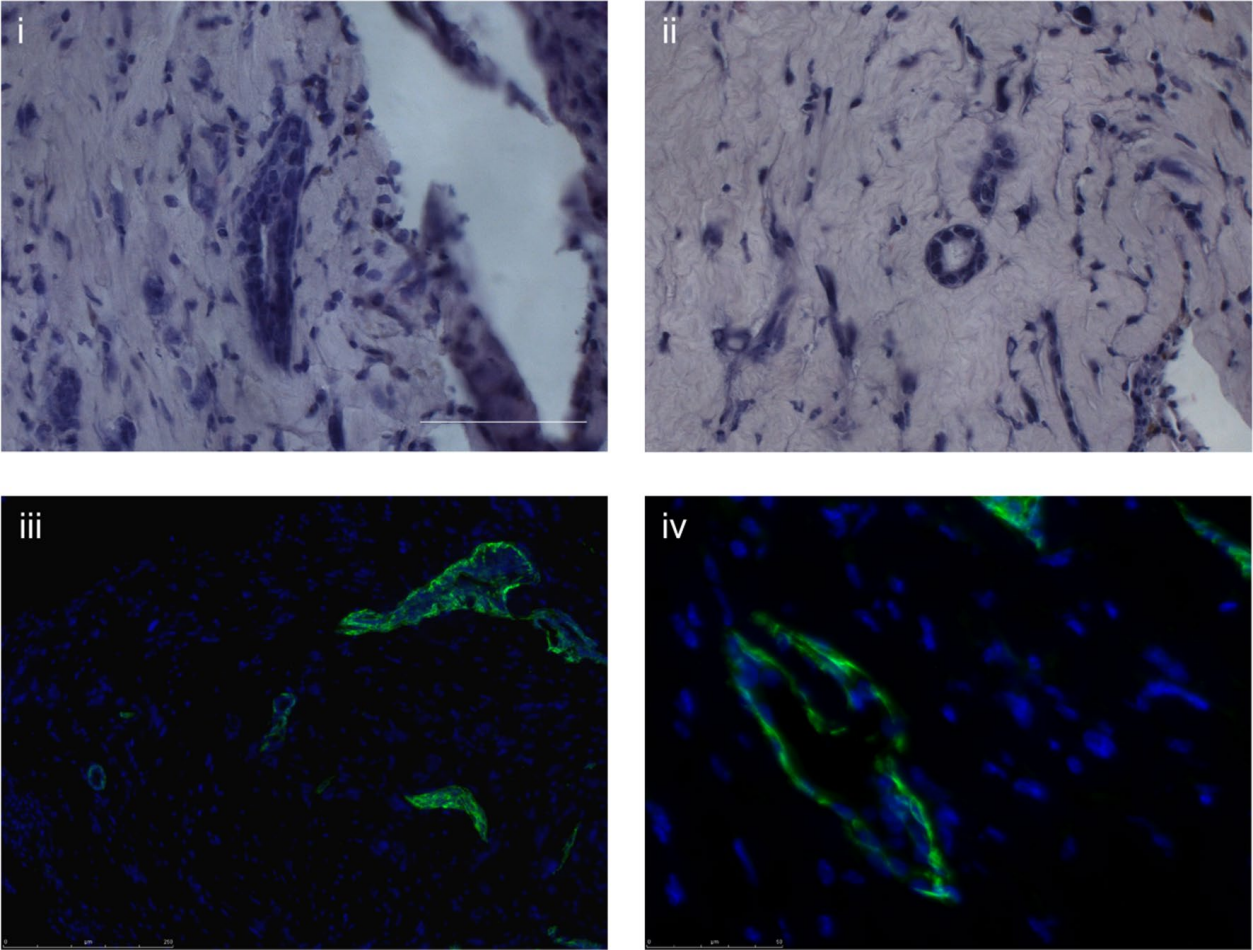
Regenerated structures in CD49f^high^/P-Cadherin^neg^ seeded gels. (**i**) and (**ii**) show representative images of regenerated outgrowths in which an inner lumen and a double layer is visible. Hematoxylin staining, scale bar is 50 µm. (**iii**) and (**iv**) shows immunofluorescence staining for cytokeratin (CK) 14. CK14 is in green, nuclei are stained blue with DAPI, scale bar is 50 µm.

After inclusion and sectioning of the gels, outgrowths were detected only in those seeded with the CD49f^high^/P-Cadherin^neg^ fraction (*n* = 9) (Fig. 3). The regenerated structures exhibited an internal lumen. Both round-shaped and slightly elongated outgrowths were visible even in the same section and consisted of an inner layer of cuboidal cells and an outer layer of flat, more elongated CK14^+^ cells (Fig. 4). Occasionally, multilayered structures could be detected. In gels seeded with cells from the other fractions (*n* = 9 for each subpopulation), no outgrowths could be detected.

By dissociating the recovered gels and seeding the cells in a CFC assay, evaluation could be undertaken of whether committed progenitor cells were produced by stem cells during the xenograft. Only CD49f^high^/P-Cadherin^neg^-engrafted cells were able to produce distinct colonies. Such colonies included myoepithelial-like (with a frequency of 36.7 ± 1.8%) and luminal-like ones (63.3 ± 6.9%) (Fig. 2ii).

### Whole transcriptome analysis of mammary subpopulations

The first step in the analysis of the whole transcriptome of the four different populations consisted of fitting the mixed model. Since normalization of arrays occurred prior to analysis, no significant effect of cell population is expected, as was observed here (*P* = 0.36). Also no between-sample variation is expected, as supported by the small variance component estimate ($\hat{\sigma }_{S}^{2} = 0.036 \pm 0.019$). However as expected there was substantial overall between-gene variation ($\hat{\sigma }_{G}^{2} = 5.44 \pm 0.070$), and also substantial Gene.CellPop effects ($\hat{\sigma }_{G.CP}^{2} = 0.672 \pm 0.006$) suggesting the existence of DE genes.

### Differentially expressed genes

Table 1 shows the results of fitting of the mixture model for DE and non-DE genes. Based on the model, a large proportion of genes showed evidence of differential expression from 23% of genes (CD49f^mid^/P-Cadherin^mid^) to 39.7% (CD49f^high^/P-Cadherin^neg^). Using the threshold of a probability of 0.8, individual genes were classified as DE or non-DE. The false discovery rates (FDR) of DEGs was also calculated using this threshold, for each cell population. Overall, it is estimated that 3.2% of genes identified as being DE are ‘false discoveries’. A complete list of DE genes is available in Supplementary Table 1.

**Table 1:** Results of fitting of the mixture model for DE and non-DE genes.

	Cell population			
	CD49f^neg^ P-cad^neg^	CD49f^mid^ P-cad^mid^	CD49f^mid^ P-cad^high^	CD49f^high^ P-cad^neg^
π_1_	0.227	0.225	0.307	0.375
σ_0_	0.302	0.204	0.350	0.261
σ_1_	1.290	0.612	1.119	1.109
*n*_DE_	1,433	980	1,644	2,669
FDR	0.030	0.041	0.035	0.029

π_1_ = estimated proportion of genes that are differentially expressed (DE); σ_0_ = SD of the Gene × Cell population gene effects in each cell population for the non-DE genes; σ_1_ = corresponding SD for DE genes; *n*_DE_ = number of DE genes detected; FDR = false discovery rate of a gene being DE based on a threshold probability of > 0.8.

**Figure 5:**
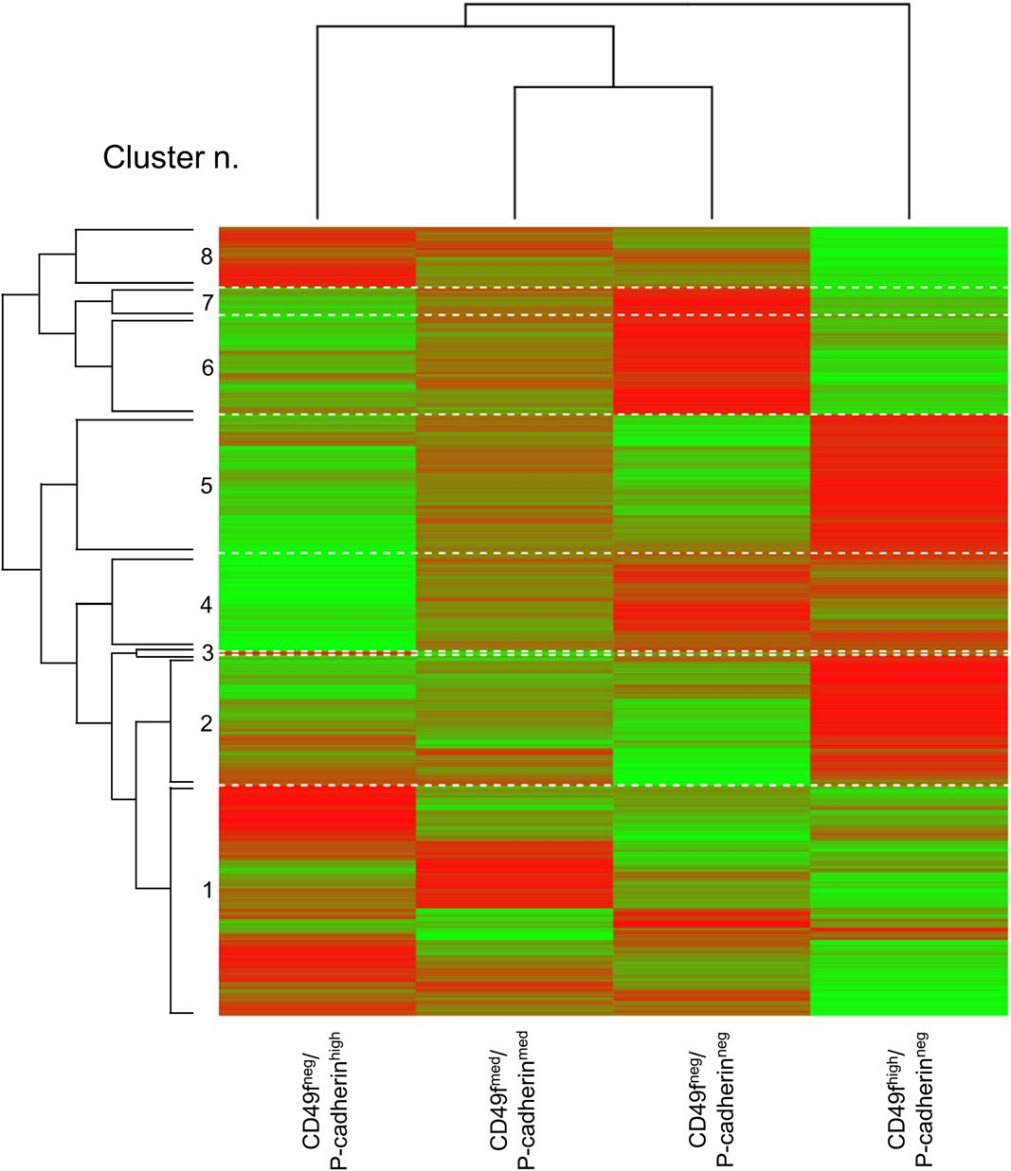
Clustering of DE genes. The graph shows the eight clusters of DE genes distributed among the four different mammary epithelial subpopulations that were used to perform the analysis for enrichment of biological processes, molecular function and cell components associated genes.

A general comparison of the four different subpopulations was initially undertaken. Genes were clustered according to their pattern of expression and a heatmap of DE genes was generated (Fig. 5). A list of DE genes split into 8 clusters (3rd level of splitting, 2^3^) was generated and a functional analysis for enrichment for biological processes (BP) was carried out. The analysis was performed both on the annotated bovine transcriptome and by translating bovine transcripts in their human orthologs.

When the bovine annotated transcripts were used, Cluster 3 showed an enrichment for transcripts associated with cell-to-cell communication (signal transduction, 68 transcripts; signaling, 71; cell communication, 71; regulation of cellular process, 92; cellular response to stimulus, 75). In Cluster 8, an enrichment in metabolic process (183 transcripts) was found. Cluster 2 showed an enrichment in genes associated with immune response and leukocytes activation although the number of transcripts per each specific category was between 3 and 9 and many transcripts were shared among the categories.

When the analysis was repeated with the human orthologs, an increased number of categories of enrichment were found. Again, in Cluster 2 biological processes associated with an immune response and activation of different leukocyte populations (in particular lymphocyte) were observed. In contrast to what was identified when using bovine annotated transcripts, Cluster 3 showed an enrichment in biological processes again associated with immune response, similar to Cluster 2. In Cluster 4, biological processes associated with angiogenesis and cell junction assembly were observed to be enriched in these transcripts. More interestingly, in the same cluster, the following biological processes could also be found: anatomical structure formation involved in morphogenesis (104 transcripts), cell migration (114 transcripts), tube morphogenesis (81 transcripts), epithelial cell migration (34 transcripts), epithelial cell differentiation (47 transcripts), epithelial cell proliferation (36 transcripts). In Cluster 8, an enrichment in transcripts was found for biological processes either associated with chromosome/chromatid segregation (20 and 14 transcripts respectively), mitotic nuclear division (21 transcripts) and DNA repair (19 transcripts), or with cofactor metabolic process (46 transcripts), oxidation–reduction process (76 transcripts), carboxylic acid metabolic process (82 transcripts), cellular amide metabolic process (50 transcripts).

When analyzing differentially expressed genes, 445 genes were overexpressed exclusively in the CD49f^high^/P-Cadherin^neg^ population while 333 were downregulated in the same subpopulation when compared to the other three (that is they were not detected as DE in the other subpopulations) (Supplementary Fig. 1).

Among the overexpressed genes, FGFR1, members of MAP kinase family (MAP3K11, MAPK11), ADAMTS1 and ADAMTS7 metalloproteases, MEF2A transcription factor, CADM4 cell adhesion molecule and several RHO GTPases (RHOB, RHOD, RHOF, RHOJ) were detected. Notably, Fos was among the downregulated gene along with HOXC9.

The same functional analysis for enrichment was subsequently performed on these two gene subsets. Only the analysis on the human orthologs was performed for better annotation.

At first an analysis for enrichment of biological process-related genes was performed. No functional enrichment was found when the analysis was restricted to the subset of the upregulated genes. However, in the subset of the downregulated genes, an enrichment for genes associated with chromosome organization, mitotic cell cycle, mitotic cell cycle process and sister chromatid segregation was found (Supplementary Fig. 2i). When single genes in these enrichment categories were examined and their specific function investigated in the literature, many were related to chromatin condensation, kinetochore formation, histone acetylation and cell-cycle progression checkpoints.

We then performed an analysis for enrichment of molecular function (MF)-related genes. In the subset of the upregulated genes, we could detect specific enrichments for genes associated with the cadherin binding and cell adhesion molecule binding functions (Supplementary Fig. 2ii). When single genes in these categories were examined and their function investigated in literature, most of them promoted cytoskeleton organization, cell adhesion, cell migration and focal adhesion. No statistically significant enrichment was found for MF in the downregulated subset of genes.

## Discussion

This study reports for the first time the utility that P-Cadherin may have in the purification of adult stem cells of the mammary gland in the bovine species. P-Cadherin has been observed to be crucial for preserving the structural integrity of epithelia in general^22^ and for maintaining important functions related to cellular differentiation and polarity, cell growth and cell migration also in adult tissues, although its role has proved prominent mostly during embryonic development^25,39^.

At the level of the mammary gland it has been reported that P-Cadherin is co-expressed with markers of the basal layer of the mammary alveolus in the mouse in association with CK5 and CD49f^40^ but it has not been investigated in more detail if it has a relevance for the regulation of the stem and / or progenitor cells. Our results propose that P-Cadherin may have an influence in cellular differentiation and its segregation outside a progenitor-rich compartment is important for the characterization of stem cells in the basal mammary phenotype when analyzed along with CD49f, a recognized marker of mammary stemness.

Our study reports that mammary stem cells exhibit a cellular phenotype (CD49f^high^ /P-Cadherin^neg^) which identifies a subpopulation of cells capable of regenerating polarized mammary acini unlike the other three subpopulations sorted with different levels of co-expression of the two membrane markers.

The overall frequency of colonies was lower than 0.05% in the double negative cells, while when cells of the other fractions were seeded, it ranged from 2.7 to 4.1%. Such frequencies would exclude the presence of progenitors in the double negative compartment, while indicating their presence in the other three. While the overall frequency of progenitor cells did not differ among the three subpopulations, it is important to emphasis that colony types differed among the remaining cell fractions. Interestingly, the analysis of cellular colonies resulting from the clonal proliferation of these populations shows the capacity of the CD49f^high^/P-Cadherin^neg^ population to differentiate into luminal-only colonies and basal-only colonies as demonstrated by expression of markers that we previously reported^16,33^. In fact, both in the CD49f^mid^/P-Cadherin^mid^ and in the CD49f^mid^/P-Cadherin^high^ fractions, no myoepithelial-like colonies could be found and only luminal-like colonies were apparent. In the CD49f^high^/P-Cadherin^neg^ fraction however, myoepithelial-like colonies could be detected with relative high frequency (71%). As for the out-growths, only CD49f^high^/P-Cadherin^neg^-engrafted cells were able to produce colonies at all. Moreover myoepithelial-like and luminal-like colonies were both evident. Such behavior adds proof to the stem identity of the engrafted cells from the CD49f^high^/P-Cadherin^neg^ subpopulation.

The organization of the mammary stem niche in the bovine appears, as previously reported, more similar to the mammary stem niche of the humans rather than the murine^8,33^. The observed prevalence of mixed colonies in assays of cells isolated from regenerated structures is similar to what we have seen in similar assays of human cells and likely reflects the early regenerative phase of the structures from which they were obtained. In addition, the niche requirements of these primitive bovine mammary stem cells appear to more closely resemble those of their human rather than their murine counterparts since they regenerated structures in the kidney capsule assay but not when injected directly into cleared mouse mammary fat pads^3,41^.

Higher expression of P-Cadherin seems to be restricted to luminal-only progenitors and therefore might play a role in luminal differentiation in adult mammary tissue. This observation has been reported in human cancer cell lines^28^ that underlined the correlation with ALDH1, a well-known cancer stem cells marker^42^ and an association with a phenotype of luminal progenitor more than stem cells. In our study a specific functional test for stem cells relate strictly the absence of P-Cadherin to a normal primitive niche. The CD49f^mid^/P-Cadherin^mid^ and the CD49f^mid^/P-Cadherin^high^ fractions do not give rise to myoepithelial colonies when seeded at clonal density. Such observations lead us to hypothesize that P-Cadherin expression becomes relevant when stem cells have already started differentiating towards the phenotype of the innermost layer of the alveolus, which is formed by secretory epithelial cells. Previously, similar observations have always been reported in the bovine for E-Cadherin for its co-expression with the luminal phenotype^14^. However, we report here that P-Cadherin absence is associated with the maintenance of a stem state.

Some authors have reported that mammary stem cells are associated with a high expression of CD49f^43^. Therefore, separating different mammary subpopulations just by using CD49f can result in an enrichment in the stem cell fraction by selecting for the cells with the highest expression of this marker. However, such sorting strategy would suffer from a major drawback, specifically the variability in setting a gate for such a population. The use of a secondary marker, such as P-Cadherin, allows for a better separation of mammary subpopulations since P-Cadherin expression is different in the CD49f^mid^ and CD49f^high^ populations. This approach results in a higher purity of the mammary stem cell population. The similarity with the behavior reported above with the stem niche characterized for the human species allows us to hypothesize that this surface marker in association with CD49f increases the purity of cellular subpopulation for stemness.

The transcriptome analysis of the different cell subpopulations highlights that P-Cadherin^neg^ cellular subpopulation has a downregulation of genes related to the progression through cell cycle, which is consistent with a quiescent nature of adult stem cells. We have reported that 445 genes were overexpressed in the stem-cell enriched mammary subpopulation identified in CD49f^high^/P-Cadherin^neg^ phenotype while 333 were downregulated in the same subpopulation. In particular, in the subset of the downregulated genes, an enrichment for genes associated with chromosome organization (such as SMC2), mitotic cell cycle (such as CDC23), mitotic cell cycle progress, sister chromatid segregation (such as SYCP3, KIF22 and AURKB), chromatin condensation, kinetochore formation (such as KIF2C and CENPQ), histone acetylation (such as EYA3 and HDAC11), and cell-cycle progression checkpoints (such as CCNE1 and MYB) was observed. It was reported that some types of stem cells, such as long-term hematopoietic stem cells and skeletal muscle stem cells, are characterized by prolonged periods of quiescence^44,45^. The identity of the niche signals required for long-term stem cell quiescence, and the mechanisms that underlie the transition from quiescence to activation, are not completely understood however interesting results have been proposed^46^ N-Cadherin and M-Cadherin have been proposed as niche-based regulators of SC quiescence assuming that cell–cell adhesion exerts a key role in niche-regulated stem cell behavior, with cadherin-based adhesive junctions anchoring stem cells to niche cells^47^. In our study, a correlation for the activation of the mammary stem niche could be hypothesized due to the presence of P-Cadherin which would initiate a partial differentiation towards cellular precursors. In these mammary epithelial subpopulations that lack the expression of P-Cadherin we show that genes related to cell cycle are downregulated while genes related to cytoskeleton organization are upregulated, thus when this cadherin is expressed the cells loose the focal adhesion, start to migrate from their stem niche and proliferate.

In conclusion, this study reports for the first time the role of P-Cadherin to isolate the adult mammary stem cells subpopulation at higher purity in association with CD49f, indicating how the expression of this cadherin is important for the recruitment towards a more differentiated phenotype and, as already reported for other cadherins, its absence is linked to the activation or shutdown of genes associated with cell quiescence. This observation could be useful for identifying new strategies for controlling stem cell mobilization in mammary tissue.

The data discussed in this publication have been deposited in NCBI's Gene Expression Omnibus^48^ and are accessible through GEO Series accession number GSE148447 (https://www.ncbi.nlm.nih.gov/geo/query/acc.cgi?acc=GSE148447.

## Materials and methods

### Tissue collection and cell isolation

The study on cows/animals were carried out in accordance with relevant guidelines and regulations: whole udders were collected from Piedmontese breed cows (age ranging from 17 to 28 months) at a local abattoir within 2 h of the time of slaughter. Sample collection, after the slaughter, was performed with the authorization and under the supervision of representatives of the Veterinary Services of the Italian National Health Service branch of the Ministry of Health. Animal work described in this study (NOD-SCID mice experiments) has been reviewed and approved by the Italian Ministry of Health (Authorization note no. 149401894128) and by the Bioethics Committee of the University of Turin.

Mammary tissue was then processed as previously described^33^ in order to obtain a single cell suspension to be used for subsequent assays. Briefly, tissue samples were dissociated overnight in Dulbecco’s Modified Eagle’s Medium/Nutrient Mixture F12 Ham (DMEM/F12) 1:1 v/v mixture supplemented with 2% bovine serum albumin (BSA), 300 U/ml collagenase type IV, 100 U/ml hyaluronidase type IV-S, 100 U/ml penicillin and 100 μg/ml streptomycin (all from Sigma-Aldrich Corp., St. Louis, MO, USA).

Epithelial aggregates were then separated by differential centrifugation and enzymatically digested with a 0.5 mg/ml trypsin solution supplemented with 0.2 mg/ml EDTA followed by a digestion with 5 mg/ml dispase and 100 µg/ml DNAseI (all from Sigma-Aldrich Corp.).

### Flow cytometry and sorting

After the dissociation of the epithelial organoids, a single cell suspension was obtained. The cell suspension was then further filtered through a 40-µm mesh. Cells were then counted, centrifuged and resuspended in a suitable volume of Hank’s Balanced Salt Solution (HBSS) supplemented with 2% Fetal Bovine Serum (FBS, Gibco, Thermo Fisher Scientific, Waltham, MA USA). Cell concentration was kept lower than 0.5 × 10^7^ cells/ml. Antibodies were then added to the cell suspension at the following concentration: 5 µl/10^6^ cells CD49f.-PE (clone GOH3, Santa Cruz Biotechnology, Dallas, TX USA), 10 µl/10^6^ cells P-Cadherin-FITC (N-19, Santa Cruz Biotechnology), 20 µl/10^6^ cells CD45-Alexa647 (CACTB51A, Monoclonal Antibody Center—Washington State University, Pullman, WA USA that was conjugated with Alexa647 using an APEX Antibody Labeling kit from Thermo Fisher Scientific). Cells were incubated for 20 min at 4 °C in the dark and then washed with HBSS supplemented with 2% FBS and resuspended in a suitable volume of HBSS. Immediately before sorting 7-Aminoactinomycin D (7-AAD) was added to each tube for live/dead cells discrimination at a final concentration of 1 µg/ml. 7-AAD negative events were selected to exclude dead cells and CD45 negative events to exclude immune cells. 7-AAD^-^/CD45^-^ cells were then sorted according to the expression of CD49f. and P-Cadherin by use of a FACSAria III (BD-Becton Dickinson, Franklin Lakes, NJ USA) equipped with an 85-µm nozzle tip and three lasers (405, 488 and 633 nm).

### Colony forming cell (CFC) assay

CFC assays were carried out as previously described^33^. Briefly, single cell suspensions of bovine mammary epithelial cells were prepared from the sorted fractions and seeded at very low density (2 × 10^3^ cells per 60-mm dish) along with 2 × 10^5^ NIH 3T3 fibroblasts that were previously treated with mitomycin C (10 μg/ml for 2 h).

Cells were cultured for 24 h in human EpiCult B medium (STEMCELL Technologies, Vancouver, BC, Canada) supplemented with 5% fetal bovine serum (FBS) and 10^-6^ M hydrocortisone, 100 U/ml penicillin and 100 μg/ml streptomycin (Sigma-Aldrich Corp). Subsequently the medium was replaced with fresh media but without FBS and cells were cultured for seven additional days.

Colonies were then examined under a Leica DM IL inverted contrast phase microscope in order to assess their morphology and scored accordingly.

### Xenograft

Immediately after sorting, cells from each fraction were resuspended and mixed with 10T1/2 mouse fetal fibroblasts that were previously treated with mitomycin C at a concentration of 2 μg/ml for 16 h. Cells were then embedded in rat tail collagen as previously described^33^ and transferred under the kidney capsule of female NOD/SCID mice. Mice received a silicone pellet (MED-4011, NuSil Technology, Carpinteria, CA, USA) containing 2 mg of 17β-estradiol and 4 mg of progesterone (both from Sigma-Aldrich Corp) at the time of surgery. The gels were recovered 4 weeks later.

### Staining of paraffin embedded sections

When gels were recovered from mice, they were processed as previously described for paraffin embedding^33^. Briefly, gels were fixed in 4% neutral buffered formalin overnight and subsequently dehydrated and paraffin embedded. Sections of 4 µm were cut and processed for either hematoxylin eosin staining or for immunofluorescence staining.

After dewaxing, sections were placed in Tris–HCl buffered saline supplemented with Tween-20 (TBS-Tween, 0.1 M Tris HCl, 0.14 M NaCl, 0.05% Tween-20, pH7.6, all reagents from Sigma-Aldrich) for 10 min, then incubated at room temperature for 1 h in TBS-Tween supplemented with 10% goat serum (Sigma-Aldrich) and then for 1 h at room temperature with the primary antibody. Sections were then washed 3 times with TBS-Tween, the secondary antibody applied and incubated for another hour. Sections were then counterstained with 4′,6-diamidino-2-phenylindole (DAPI, Sigma-Aldrich) and then mounted.

As primary antibody, human cytokeratin 14 (CK14, 1:500 dilution, polyclonal AF-64, Covance, Princeton, NJ, USA) was used, while as secondary antibody, AlexaFluor 488-labelled goat anti-rabbit IgG was used (1:500, Invitrogen, Thermo Fisher Scientific).

### RNA extraction and sequencing

In order to perform a whole transcriptome analysis, total RNA was extracted from cells of the four different fractions. Cells were directly sorted in 1.5 ml tubes containing 1 ml of TRI Reagent (Sigma-Aldrich Corp). For each fraction, at least 5 × 10^5^ cells were collected and lysed for RNA extraction.

RNA was purified by adding 200 µl of chloroform (Sigma-Aldrich Corp.) and centrifuging at 12,000×*g* for 15 min at 4 °C. The upper clear phase was recovered and RNA was precipitated with 500 µl of isopropanol (Sigma-Aldrich Corp.) followed by a wash with 70% ethanol (Sigma-Aldrich Corp.). The RNA pellet was then resuspended in DEPC water (approximately 20 µl) and quantified with a Nanodrop 2000 (Themo Fisher Scientific).

RNA samples were then shipped to IRCCS Ospedale San Raffaele, Italy, where they were processed for an Illumina TruSeq sequencing protocol with a reads depth of 30 M and expression data were normalized as RPKM.

### Gene expression analysis

The data set allowed to compare patterns of gene expression across the four cell types, namely CD49f^−^/P-cad^-^ (*n* = 3); CD49f^+^/P-cad^+^ (*n* = 2); CD49f^++^/P-cad^-^ (*n* = 3); and CD49f^+^/P-cad^++^ (*n* = 3). Data analysis was conducted by using a two-stage approach, as outlined by Singh et al.^24^ and Trabzuni et al.^34^. Firstly, a large-scale linear mixed model was fitted to all the gene expression data, of the form

$$ {\text{logExpr}} = {\text{constant}} + {\text{CellPop}} + {\text{Sample}} + {\text{Gene}} + {\text{Gene}}.{\text{CellPop}} + \varepsilon $$

where logExpr is the logarithm of the expression value, CellPop is the fixed effect of the cell population type, Sample is the random effect of the array, Gene is the random effect of a particular gene, and Gene.CellPop is the specific random effect of a gene in a particular cell population, and ε is the random error. Of main interest it will be the estimates of the Gene.CellPop terms. Fitting of the linear mixed model was conducted using ASReml-R^35^ within the R computing environment.

The second stage of the analysis involved fitting a two-component mixture model for these Gene.CellPop effect estimates, separately for each cell population. The two components are a set of differentially expressed (DE) and non-differentially expressed (non-DE) genes. Genes are assigned as DE when the (posterior) probability of being DE exceeds 0.8.

Following this, some descriptive approaches were used, particularly to investigate patterns of differential expression across the four cell population types. All analyses were conducted using R.

### Gene annotation and functional analysis

Genes named after their ENSEMBL ID have been translated to their common gene name in order to have the same identifier for all genes considered, the translation has been run with data from BioMart tool as in Ensembl Release 96 (April 2019) based on the bovine genes ARS-UCD1.2 assembly.

Gene ontology enrichments and gene functional analysis have been conducted in R environment, release 3.6.1, through Bioconductor (https://www.bioconductor.org/) package ClusterProfiler, version 3.12.0, a 0.05 cutoff value has been chosen for false discovery rate values. Bovine and human functional annotation were based on org.Bt.eg.db^B^ and org.Hs.eg.db^C^^36^, respectively, and homologene^D^^37^ package has been used for cow-human gene orthology conversion.

## Supplementary information

Supplementary file1

Supplementary file2

Supplementary file3
