# Supplementary material for: Whole transcriptome analysis of bovine mammary progenitor cells by P-Cadherin enrichment as a marker in the mammary cell hierarchy
Source: Sci Rep. 2020 Aug 25;10:14183. doi: 10.1038/s41598-020-71179-4 (PMC7447765; doi:10.1038/s41598-020-71179-4)
Supplement: Supplementary file 2 — Supplementary file2 [file 41598_2020_71179_MOESM2_ESM.docx]

**Whole transcriptome analysis of bovine mammary progenitor cells by P-Cadherin enrichment as a marker in the mammary cell hierarchy**

Martignani E^1^, Ala U^1^, Sheehy PA^2^, Thomson PC^3^, Baratta M^1^*

1 Department of Veterinary Science, University of Turin, Via Largo Braccini 2, Grugliasco (TO), 10095 Italy

2 Sydney School of Veterinary Science, The University of Sydney, 425 Werombi Road, Camden NSW 2570, Australia

3 School of Life and Environmental Sciences, The University of Sydney, 425 Werombi Road, Camden NSW 2570, Australia

**Supplementary data**

**
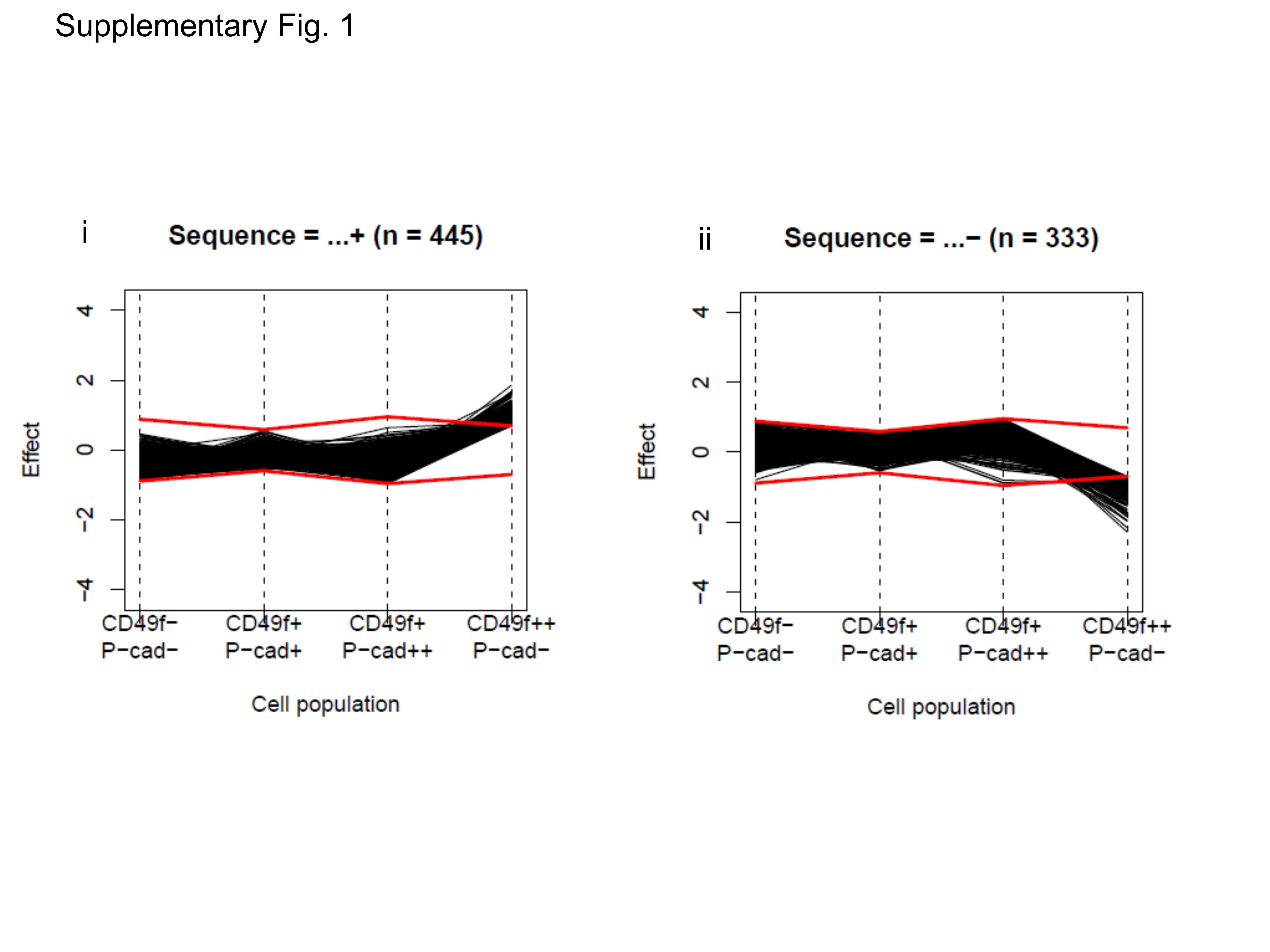
**

**
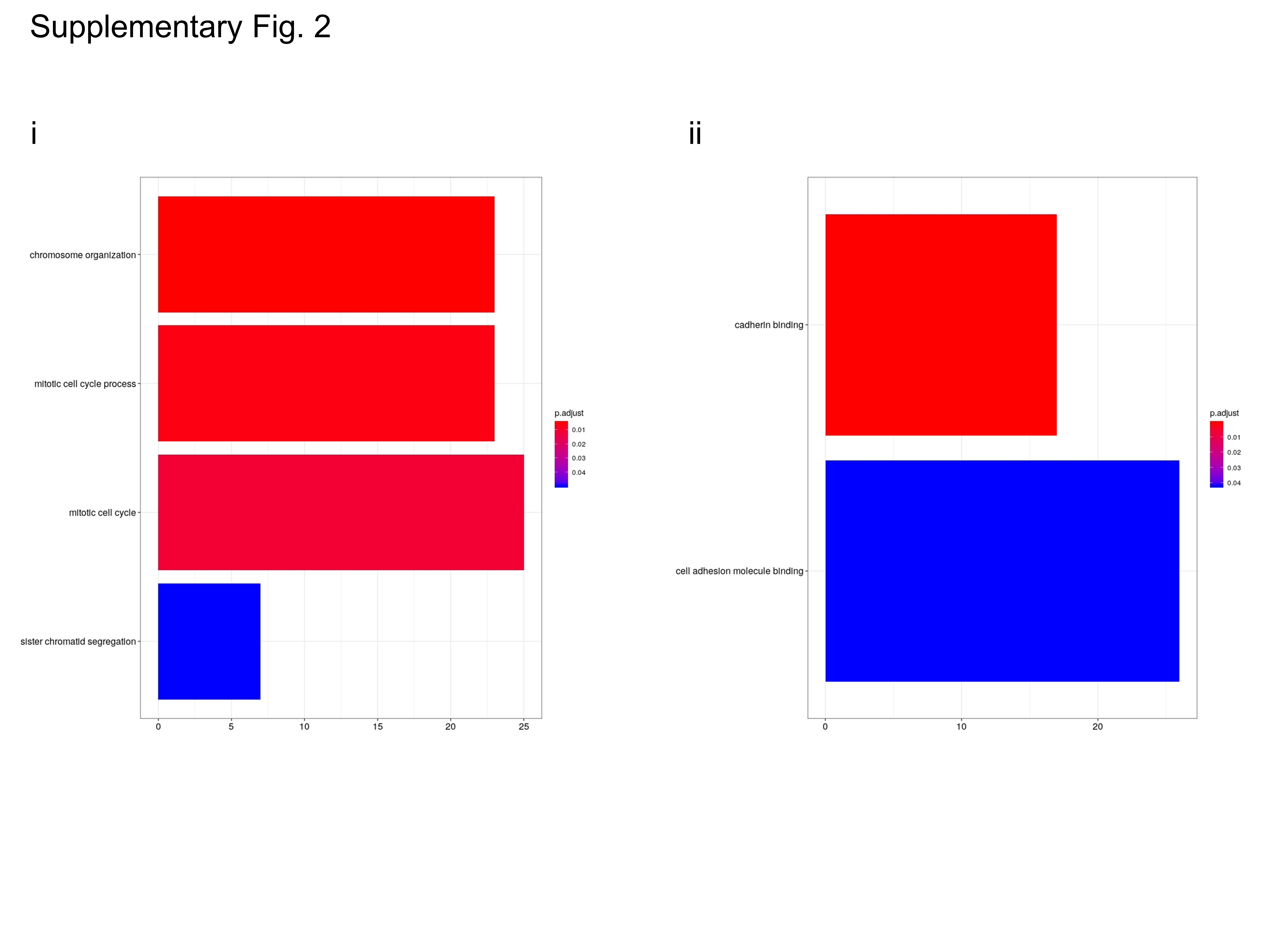
**
