# Supplementary material for: Whole transcriptome analysis of bovine mammary progenitor cells by P-Cadherin enrichment as a marker in the mammary cell hierarchy
Source: Sci Rep. 2020 Aug 25;10:14183. doi: 10.1038/s41598-020-71179-4 (PMC7447765; doi:10.1038/s41598-020-71179-4)
Supplement: Supplementary file 3 — Supplementary file3 [file 41598_2020_71179_MOESM3_ESM.docx]

**SUPPLEMENTARY MATERIAL**

**Supplementary Figure 1 Selection of DE genes associated with a mammary stem cell phenotype.** Graphs showing the expression profile of DE genes that are upregulated (i) or downregulated (ii) in the CD49f^high^/P-Cadherin^neg^ cell fraction.

**Supplementary Figure 2** Enrichment analysis for biological processes related and molecular functions related differentially expressed genes. The graphs show the biological processes enriched categories for upregulated DE genes in the CD49f^high^/P-Cadherin^neg^ subpopulations (i) and the molecular functions enriched categories for downregulated DE gene in the CD49f^high^/P-Cadherin^neg^ subpopulations (ii)

**Supplementary Table 1 Complete list of DE genes in the for mammary epithelial subpopulations.** The supplementary spreadsheet lists all of the genes that were identified as DE in the four bovine mammary epithelial subpopulations. Values in the table represent the (posterior) probability of the gene being DE in that subpopulation; genes with probabilities greater than 0.8 are considered DE in that subpopulation.
